# Psychic Life-Biological Molecule Bidirectional Relationship: Pathways, Mechanisms, and Consequences for Medical and Psychological Sciences—A Narrative Review

**DOI:** 10.3390/ijms23073932

**Published:** 2022-04-01

**Authors:** Anna Giulia Bottaccioli, Mauro Bologna, Francesco Bottaccioli

**Affiliations:** 1 Department of Psychology, University “Vita e Salute”, San Raffaele, 20132 Milan, Italy; 2 Italian Society of Psycho-Neuro-Endocrine-Immunology (SIPNEI), 00195 Rome, Italy; mauro.bologna@univaq.it (M.B.); francesco.bottaccioli@gmail.com (F.B.); 3 Department of Medicine, Public Health, Life and Environmental Sciences, University of L’Aquila, 67100 L’Aquila, Italy

**Keywords:** psychoneuroendocrinoimmunology, HPA axis, mental disorders, positive emotion, chronic stress, molecular consequences

## Abstract

Today, it is possible to investigate the biological paths and mechanisms that link mental life to biological life. Emotions, feelings, desires, and cognitions influence biological systems. In recent decades, psychoneuroendocrinoimmunology research has highlighted the routes linking the psyche–brain–immune systems. Recently, epigenetics research has shown the molecular mechanisms by which stress and mental states modulate the information contained in the genome. This research shapes a new paradigm considering the human being as a whole, integrating biology and psychology. This will allow us to progress towards personalized precision medicine, deeply changing medical and psychological sciences and clinical practice. In this paper, we recognize leading research on both bidirectional relations between the psyche–brain–immunity and molecular consequences of psychological and mental states.

## 1. Introduction

William James, the pioneer of psychological science, in *The Principles of Psychology* (1890), wrote that “no mental modification ever occurs which is not accompanied or followed by a bodily change”. Psychology, therefore, “must take account not only of the conditions antecedent to mental states, but of their resultant consequences as well” [1]. According to psychoneuroendocrinoimmunology research, emotions and feelings influence biology because the psyche and biological systems have bidirectional connections [2].

Today, we have evidence that there is communication between the neuroendocrine system and the immune system even in simple organisms, such as invertebrates [3]. Physiology, at every level of life’s evolution is integration. Without integration between systems, there is no life, at any level, from the simplest organism to the most complex one. In human physiology, this integration becomes even wider [4], as the levels of integration between biological systems also include psychic regulation. Mental processes, which the psychological and behavioral sciences summarize in the concept of “motivation”, influence the immune system and, in turn, are influenced by its activity [5]. As we see in this article, exposure to emotional stress, loneliness, depression, social withdrawal, and other mental states increases the inflammatory activity of the immune system. Conversely, inflammation, which from the immune system reaches the brain or is produced by immune cells that are an integral part of brain tissue, increases the brain’s sensitivity to different life experiences [6]. The consequence of this is that motivational processes can be immersed in an inflammatory brain matrix, causing dysfunctional behavior and psychic suffering, from which real psychiatric disorders may also arise [7,8].

## 2. Psyche and Biological Systems: The Bidirectional Pathways

### 2.1. What Is the Psyche?

The psyche can be defined as all mental activities, both conscious and unconscious. While the concept of consciousness is intuitive, although the mechanisms that generate it are still partially unknown [9,10] and its origin philosophically debated [11], the unconscious has been questioned for a long time. Today, Freudian unconscious is still the object of radical criticism and for decades has been the main target of cognitivist criticism towards psychoanalysis; the mind, according to classical cognitivism, is defined as rational. If decisions, thoughts, and behaviors are not rational, the cause lies in the improper use of mental procedures [12]. However, in the first half of the last century, a line of experimental research was conducted that shows how irrational cognitive processes are separate from the dominion of consciousness and how much they are dependent on automatic mechanisms [13,14]. Experimental psychology studies over the last 60 years have unarguably shown that human cognitive abilities, based on rational; therefore, conscious processes are very limited and only occasionally used. For the most part, they live together in unconscious and automatic ways, for example, optical illusions, false memories, conditioning, mental shortcuts (heuristic), and the conditioning of social and communication context (priming) [15].

With regard to the unconscious, it therefore seems possible to conclude that, while the Freudian idea derived from Plato and Nietzsche of the unconscious as “id”, an entity beyond our control, “a melting pot of bubbling feelings” [16] dominated by libido and aggression, has no scientific evidence, it seems to be that the human psyche uses unconscious modes of functioning that constitute the personality and emotional style of each of us. Emotions and feelings, according to Antonio Damasio [17], are a “hybrid”, sharing body and mind, and are largely unconscious, yet they are the root of consciousness. Regarding the relations between conscious and unconscious thinking, the Nobel Laureate in Physics Giorgio Parisi states that unconscious thinking is crucial for the emergence of new ideas and for mathematical intuition [18].

The psyche is, therefore, a wide system arising from the activity of the brain and body networks [19], which at the same time is able to feed back on the biological systems that support it to shape their functions [20].

The psychic world has a personal dimension, which is nourished by a persistent autobiographical narration and gives us a sense of inner unity and, at the same time, of similarity and difference with other human beings. Throughout the history of psychology and neuroscience, this has been referred to by various terms: ego, self, higher consciousness, etc. Our self is the mental space where the signals from the environment, from the intersubjective and internal relational matrix, are continuously processed, and where, to use Joseph LeDoux’s metaphor [21], our working memory “cooks emotions” and transforms them into feelings that orient thoughts, as well as emotional, motivational, and behavioral states. The processing of emotions is the product of more than one component: the activity of survival circuits, feedback from the body, attention and the labelling of signals, and an evaluation through cognitive memory (frame of mind) and autobiographical memory. This continuous mental work structures our emotional styles.

Individual emotional style, combined with personal culture and degree of adaptation to the social environment, defines our personality, which is, therefore, the combination of many factors, including what Carl Gustav Jung [22] called the “persona”, understood as the social mask that each of us wear in the different contexts in which we live. Other scholars have defined this variety of social and internal dimensions that we experience as the plurality of the states of self.

The self, therefore, is the place of constant integration, and of the continuous search for balance within intrapsychic dynamics that, in the course of individual development, are structured with a high degree of self-feeding. The level of self-integration, which Aaron Antonovsky, a psychologist and health care system scholar, called the “sense of coherence”, is relevant to the individual’s health [23]—health that the philosopher Hans-Georg Gadamer highly effectively defined as “a state of intrinsic adequacy” [24].

To conclude: (a) the psyche includes all mental activities, conscious and unconscious; (b) the psychic world has a personal dimension, which has been referred to by various terms: ego, self, higher consciousness, etc.; (c) regardless of the names used, it is a place of constant integration, and of the continuous search for balance within intrapsychic dynamics that, in the course of individual development, are structured with a high degree of self-feeding; (d) the psyche arises from the biological network and takes shape from the private and socially determined history of the individual; and € at the same time, it influences the individual biological organization, as exemplified later in this paper. This possibility of reciprocal influence is the result of the co-evolution of biological organization and psychic functions—no mind, no human brain, and vice versa.

The evolution of the brain and body, and of the mind, was a single process [10], in which psychic functions played a fundamental role in adapting the organism’s biological and physical to the environment. This is particularly evident in the most recent, and in our opinion convincing, reconstruction of the emergence of articulated language in Homo sapiens, where the modification of the skull and the appearance of the supralaryngeal vocal tract allow the emergence of linguistic functions, which in turn induces an increase in the cerebral areas of language [25,26]. The coevolution of brain size and culture and sociality has been documented in primates [27] and computer simulations [28].

Particularly in higher organisms, adaptation to the environment is driven by the construction of mental models of the world that transcend immediacy and allow predictions by modifying physiological conditions. These mental models are built from the earliest stages of life and, over the course of life, become largely automatic; however, they are modifiable, and these modifications can also an modify aspects of biology, as we see later. Here, we want to emphasize that the reductionist paradigm that crushes the psychic dimension onto the cerebral one, however canceling it, does not explain real human functioning. Similarly, the invocation of a “ghost in the machine”, to explain the influence of the psyche on the organism, cannot be scientifically accepted. Without wishing to simplify a prolonged philosophical debate on the subject, we believe that there is no gap between the psyche and biology. The two dimensions of the living human organism have evolved in Homo sapiens and develop in the individual together. For this reason, it is philosophically plausible they can communicate bidirectionally. The scientific evidence shown in this review provides valid support for the philosophical paradigm indicated above. However, we believe that there is still a long way to go in both psychological and biological disciplines. We summarize these considerations in the Conclusions section.

### 2.2. Pathways

**Figure 1:**
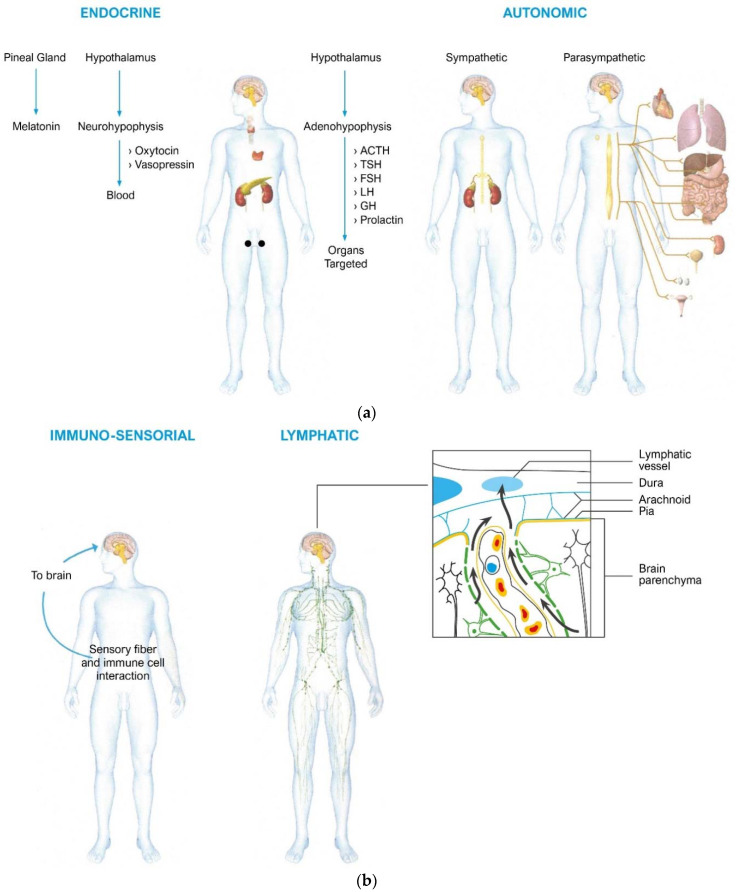
(**a**,**b**). The complex neuroendocrineimmune network. In (**a**), the endocrine pathways are indicated: the pineal gland that produces melatonin with effects of regulation of the circadian rhythms and also of the immune system (not shown); the hypothalamus which activates the neuroendocrine axes, including the stress axis; and the autonomic nervous system that influences organs and tissues with the sympathetic and parasympathetic arms. In (**b**), the sensory nervous system and immune cells communicate with each other and with the brain through the release of cytokines and neuropeptides; the figure also indicates the lymphatic connection which, contrary to a centennial dogma that considered the brain without lymphatic vessels, connects the brain to the general lymphatic system and, therefore, to the immune system organized in the lymph nodes.

Integration between the main systems is the basis of life. Inner dialogue is guaranteed by common connecting pathways and by shared receptors and molecules. Over the last 40 years, psychoneuroendocrinoimmunology research has produced incontestable evidence on communications between systems, breaking down the dogma of the lack of communication between the brain and immune system [29,30,31,32,33,34]. Figure 1a,b show the complex and pervasive human biological network, which includes the autonomic nervous system, lymphatic and blood vessels, and the endocrine system.

**Figure 2:**
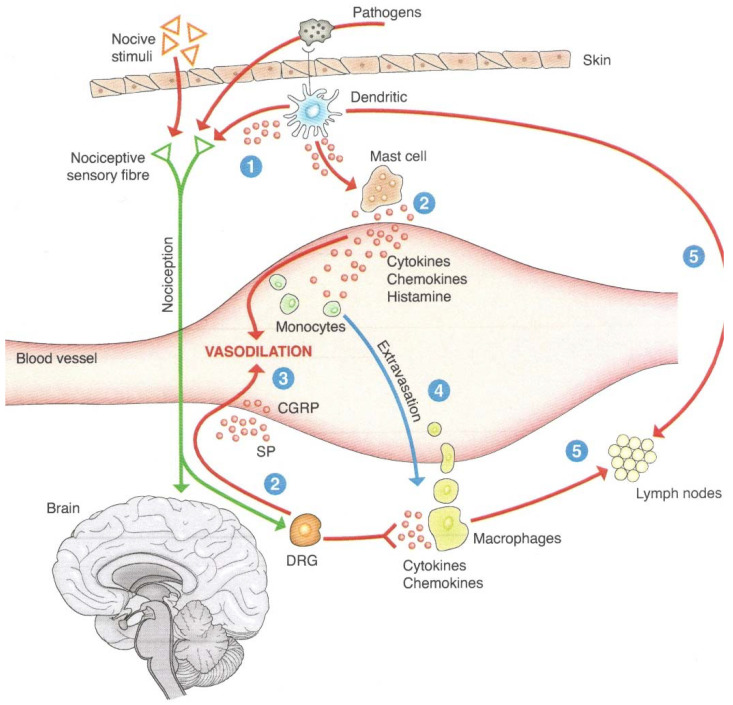
The cooperation between sensory nerves and immune cells. The figure shows (1) the shared capacity that sensory nerves and immune cells have to receive signals from pathogens, orchestrating a joint response. The sensory nerves will, on the one hand, send a pain signal to the brain (nociception) and, on the other hand (2), via the dorsal root ganglion (DRG) of the spinal cord, will produce neuropeptides (substance P (SP) and calcitonin gene-related peptide (CGRP)) that react on the blood vessel, causing vasodilation and inflammation. The same phenomena are produced by mast cell degranulation (3) with the release of inflammation mediators, including histamine. Vasodilation and the presence of cytokines and chemokines cause the circulating monocytes to be called upon, which, due to modifications of the vasal endothelium in the meantime, will be able to escape the vessel (4) by entering the tissue (represented by the skin in the figure), where they will be activated against pathogens. Finally, the activated macrophages and, in particular, dendritic cells will migrate towards the lymph nodes (5), presenting the antigen to helper T cells, completing the immune response. Source: Reprinted with permission from Ref. [2]. 2020, Edra.

To integrate the network picture, Figure 2 describes the steps that, via a harmful stimulus affecting the skin, determine the simultaneous activation of the sensory nervous fiber and immune cells (dendritic and mastoid cells). Nociceptive sensory neurons are activated both by cytokines and chemokines produced by immune cells and, according to the most recent research, by receptors capable of capturing pathogen products [35]. From this point of view, the nervous system and the immune system function as an integrated defense system, since both neurons and immune cells express receptors for pathogens and materials from dead cells, but also for signals coming from the extracellular environment, such as oxygen and acid concentration.

#### 2.2.1. From the Nervous System to the Immune System

The connection between the brain and the rest of the body is achieved through the peripheral nervous system, which is organized into somatic and neurovegetative systems that have a significant effect on vessels and immune cells. By means of neuropeptides and neurotransmitters, which are released by nerve fibers, immune response actors (lymphocytes and other immune cells, antigen-presenting cells, and vasal endothelium) receive stimulating or inhibiting inputs, which affect the onset and evolution of inflammation. In this regard, it is worth noting the anatomical connection described several decades ago by David Felten’s group, who have extensively documented the innervation of lymphoid organs, both primary and secondary [36]. The innervation of the lymphoid organs is achieved by neurovegetative fibers, mainly by releasing norepinephrine, acetylcholine, and neuropeptides. In this regard, the plexuses of sympathetic nerve fibers that envelop the arterial vessels penetrating the lymphoid organs play an important role. These fibers have a direct anatomical relationship primarily with mastoid cells, which are normally adjacent to the vasculature. These cells, which are also called mast cells, are highly inflammatory because they are able to release large amounts of active substances, such as histamine and other molecules that cause vasodilation and inflammation.

Mast cells are not only present under the skin and mucous membranes of the body, but also in fundamental organs, including the brain [37], where they can produce inflammation in connection with microglia and other brain-resident immune cells [38]. It has been shown that mast cells can be activated for inflammation by major neuropeptides (CGRP, substance P, neuropeptide Y, NGF and VIP), as well as epinephrine and norepinephrine and other substances released by nerve fibers, causing so-called neurogenic inflammation, i.e., produced directly by nerve fibers [39]. However, neurogenic inflammation seems to be regulated by other neuropeptides, including somatostatin [40] and galanin [41]. Sympathetic nerve fibers, through releasing norepinephrine and epinephrine interacting with adrenergic receptors expressed on neutrophils, monocytes, T cells, and other immune cells, regulate cytokine production and inflammation. Variable regulation is dependent on receptors. For example, anti-inflammatory effects are mediated by β2-adrenergic receptors; in contrast, α-adrenergic receptors on monocytes and macrophages can cause an increase in the production of pro-inflammatory cytokines, such as TNF-α. Cholinergic fibers releasing acetylcholine, on the other hand, can moderate inflammation; the same effects can be caused by acetylcholine-producing B e T cells [42].

Finally, in the first few years of the 21st century, a new fundamental relationship between the nervous system and immune cells was identified, which was focused on the anti-inflammatory role of the vagus nerve [43]. In the last 20 years, the research efforts aiming to explain the mechanisms by which the vagus nerve can moderate inflammation have not yet come to a definitive conclusion. The most accepted explanation involves the following circuit. For example, the afferent vagus carries an inflammatory signal intestinal TNF-α to the brain. Subsequently, a response is carried by the efferent vagus, which, not directly but through the coeliac ganglion, stimulates the spleen to produce—by means of sympathetic fibers—norepinephrine, which causes the T lymphocytes to release acetylcholine; this, in turn, by binding to a specific macrophage receptor (α7-nicotinic), inhibits the inflammatory activity of these immune cells. However, a hepatic branch of the vagus nerve has recently been identified, which directly, without the splenic nerve contribution, can moderate the experimentally induced colitis [44].

However, the vagus immunity story continues. Recent research has shed new light on neurodegenerative disorders, such as Alzheimer’s and Parkinson’s. Aggregations of misfolding proteins, amyloid beta, and tau in the case of Alzheimer’s, and alpha-synuclein in the case of Parkinson’s, are the pathogenic markers of these diseases. Experimental studies have confirmed, in the case of Parkinson’s disease, a pathogenic hypothesis advanced two decades ago by H. Braak [45], according to which misfolding of alpha-synuclein (α-syn) is formed in the gut, and subsequently, by retrograde transport and through vagal endings, reaches the brain. There is a large amount of evidence that the so-called “Lewy bodies” in the cerebral substantia nigra (pars compacta), representing a clear Parkinson’s marker, are organized around the misfolding α-syn. Such Lewy’s bodies are also present in the enteric nervous system, and through the vagus from the intestine, they can reach the brain where they follow a prion-like path of diffusion. Experiments in Parkinson’s animal models document the passage of aggregated fibrils from the intestine to the brain with death and a decrease in the number of dopamine-producing neurons in the substantia nigra pars compacta [46,47].

#### 2.2.2. From the Immune System to the Psyche–Brain System

In 1975, for the first time, Hugo O. Besedovsky showed that neuroendocrine changes take place during an immune reaction. The hypothesis was that the immune cells would initiate signals capable of reaching the brain. The hypothesis was confirmed by Besedovsky himself in 1981 and then definitively in 1986 [48].

In the years that followed, there have been extensive demonstrations that the inflammatory cytokines, IL-1, IL-6, and TNF-α, are able to induce relevant biological changes both in the main neuroendocrine axes, especially the stress axis, and in the most important brain neurotransmission systems. IL-1, in particular, is a powerful activator of the stress axis (hypothalamus–pituitary–adrenal), growth axis (GH), and prolactin, while inhibiting the thyroid and gonadal axis. At the same time, the action of cytokines, in particular IL-1, on the main neurotransmitters is documented, with an increase in metabolism and, therefore, in the consumption of norepinephrine, dopamine, and serotonin. In addition, the excitatory action of IFN-γ on the glutamate receptor, which is the most important excitatory neurotransmitter, is documented; glutamate receptor and pathway alterations are indeed thought to be at the root of all psychiatric disorders (mood disorders, psychosis, and autism spectrum disorders) and neurodegeneration. However, further confirmation of the full involvement of the immune system in disorders of the brain–psychological system is required.

**Figure 3:**
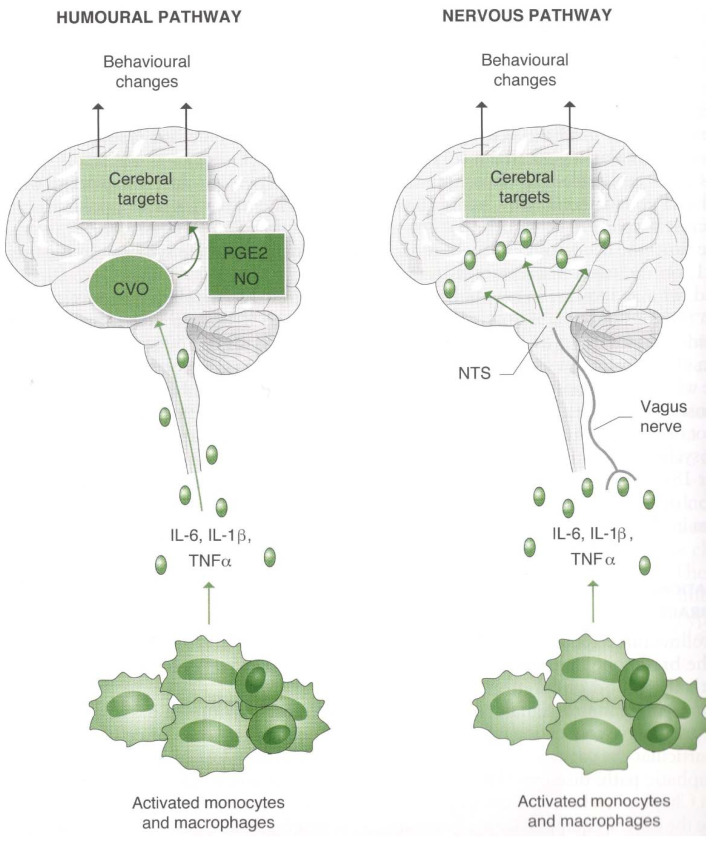
The two communication pathways from the bottom to the top: from the immune system to the brain. On the left is the humoral pathway, which, through blood circulation, carries cytokines directly to the circumventricular organs of the brain (around the ventricles) without a blood–brain barrier. Where there is a barrier, the cytokines can pass through using specific transport systems (not shown) or even by stimulating the production of other substances, such as nitric oxide (NO) and prostaglandins (PG). On the right is the nervous pathway, which—particularly through the vagus nerve, which has cytokine receptors—carries immune signals first into the nucleus tractus solitarii (NTS) and from there to the other brain structures, in particular, to the limbic system (hypothalamus, hippocampus, and amygdala). Reprinted with permission from Ref. [2]. 2020, Edra.

Cytokines follow three routes from the immune system to the brain: one humoral, carrying cytokines with the blood circulation; the second nervous, conveying immune signals to the brain through the great nerve connection routes (cranial nerves, in particular, the vagus nerve); and, finally, the third lymphatic path, which was discovered in 2015 by Antoine Louveau and coworkers [49]. Figure 3 illustrates the nervous and humoral immunity–brain communication pathways.

Cytokine signaling from the periphery to the brain is part of the more general enteroception “referred to as the process by which the nervous system senses and integrates in-formation about the inner state of the body” [50]. There are many cerebral areas, cortical and subcortical, involved in enteroception; among these, the posterior insular cortex plays a central role processing both emotional and biological signals, including aversive state [51].

There is evidence of dysregulation in insula-centered brain networks in patients with depression and particularly in patients with depression with a history of suicide attempt [52]. The insular cortex is sensitive to pro-inflammatory cytokines. Recently, researchers, after inducing intestinal inflammation, were able to map in the animal’s brain the set of neurons that traced the memory of this internal biological event. The proof of this connection came from two subsequent experiments: one showed that after the healing of the intestinal inflammatory lesion (colon and peritoneum), the stimulation of the aforementioned neuronal circuit reactivated intestinal inflammation; another showed that the inhibition of immunological brain memory reduced the extent of experimentally induced intestinal inflammation. The brain area where the memory of intestinal inflammation was located is the posterior insular cortex [53].

A relevant pathway of communication from the immune to the psyche–brain system is the bidirectional microbiota–gut–brain axis. The total number of bacteria, viruses, archaea, protozoa, and fungi in mucosae and in the skin is estimated to be of 38 trillion (3.8 × 1013) by some authors [54], while for others, it is greater by a factor of 10 (3.8 × 1014) [55]. This huge amount of microbes is present in a proportion of over 90% in the gastrointestinal tract, while a moderate number is present at the level of the stomach and the first sections of the small intestine, becoming finally more numerous around the end of the ileum and particularly in the colon (30–50% of the volume of the contents of the colon is composed of microbes, which are, to a large extent, anaerobes). It is evident, therefore, that a balance is created between the individual and these huge microbial populations in the form of a mutualistic symbiosis. We supply the food to the microbes, and they also supply us with useful substances: short-chain fatty acids (SCFA), vitamins (K and B vitamins), and neurotransmitters (noradrenaline, glutamate, and GABA). Recent research has shown that SCFAs play an important role in both the immune and psyche–brain systems. In particular, SCFA butyrate performs an epigenetic regulatory activity of inflammation (see below).

In addition, we know that balanced intestinal microbiota regulates the integrity of the mucosa, strengthening its barrier function against pathogens and stimulating a balanced immune response. Although the vast majority of gut microbial populations are not pathogenic, there are well-established groups of potentially pathogenic microorganisms that do not produce signs of alteration or disease because they are kept under control by the immune system. This set of pathogenic and non-pathogenic populations is a source of physiological stimulation of the immune system, to which it modifies itself and maintains its own balance between tolerance and reactivity. Diverse factors, namely nutritional, pharmacological, and environmental factors, can affect microbiota homeostasis, causing inflammation and intestinal dysbiosis.

**Figure 4:**
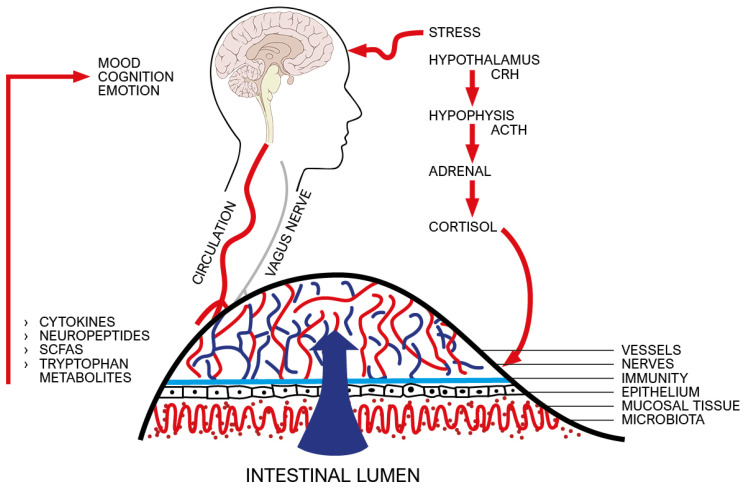
The physiological stress response is activated by physical environmental factors (such as heat and cold, humidity and drought, wind, noise, and pollutants), as well as by endogenous factors (such as a significant reduction in blood pressure and volume of circulating blood, body hydration and nutrition, as well as an infection or a hemorrhage), but also by emotional or cognitive factors [58]. The paraventricular hypothalamic nucleus (PVN) is the cerebral structure that activates the stress response. It is divided into three sectors: magnocellular, which releases oxytocin and arginine vasopressin; parvocellular, which releases corticotropin-releasing hormone (CRH); and the neurons projected towards the brain stem, where the nuclei activating the sympathetic nervous system are located. CRH, a 41 amino acid molecule, is the activator of the neuroendocrine stress pathway, with ultimate production of cortisol from the fasciculate area of the adrenal cortex. The PVN is under the control of cortical circuits, particularly of medial prefrontal cortex, and subcortical circuits, particularly of bed nucleus of the stria terminalis (BNST), hippocampus, and amygdala. Animal research and brain imaging studies in humans have documented that CRH production is controlled by activating and inhibitory circuits through the release of glutamate and GABA, respectively. PVN is directly excited by afferents from the brainstem and hypothalamic circuits, whereas afferents from the prefrontal cortex and hippocampus inhibit the HPA axis, via BNST GABA-ergic projections [59]. For four decades, the molecular mechanism by which CRH is raised under stress has been known. Activation of neural pathways afferent to CRH neurons in the PVN causes a rapid calcium influx that stimulates the fusion of CRH-containing vesicles to the cell membrane and subsequent release CRH [60]. Figure shows that emotional stress, through the cortisol release from adrenal cortex, can disrupt the intestinal barrier, causing dysbiosis and intestinal inflammation which in turn can reach the brain by altering mood and cognition. SCFA: short-chain fatty acids.

Emotional stress, via glucocorticoids and catecholamines, can disrupt the intestinal barrier, causing dysbiosis and intestinal inflammation, which in turn, via cytokines and microbiota metabolites, carried from the blood and vagus nerve, can reach the brain by altering mood and cognition, allowing the development of neurodegenerative disorders [56,57] (see Figure 4).

The dynamic equilibrium of the human network is ensured by a multiplicity of local homeostatic systems and by the central role of the brain as the coordinator of physiological and behavioral adaptation processes.

The concept of allostasis or stability through change, which was proposed in the late 1980s by Peter Sterling and Joseph Eyer [61], and in the following decades developed by Bruce S. McEwen [62], provides a useful framework for interpreting the dynamics of the organism, i.e., the systemic and molecular change it undergoes in response to the external and internal stressors. The allostasis paradigm integrates the concept of stress, developing the model originally described by Hans Selye [63]. Under stress, the organism does not strive to restore its homeostatic systems to their “normal” values of equilibrium, but implements multi-systemic and coordinated modifications, both physiological and behavioral, capable of achieving a new balance and improved fitness. However, allostatic adaptation has a cost, called “allostatic load” [64]. Under conditions of repeated or chronic stress, physiological changes become less “elastic” and not completely reversible; short-term adaptive changes, if maintained in the long-term, can wear out regulation systems and have negative consequences on the body [2].

The stress response is based on some circuits, which may be activated individually or together, depending on the type and extent of the stressor. There are three branches in the hypothalamic paraventricular nucleus: the hypothalamus–pituitary–adrenal cortex axis with the mediation of the hormones CRH-ACTH-cortisol; the hypothalamus–sympathetic nervous system–adrenal medulla axis with the production of catecholamines (epinephrine, norepinephrine and dopamine, in decreasing quantities); and the hypothalamus-neurohypophysis circuit with the release of arginine vasopressin and oxytocin.

#### 2.2.3. The Brain

Animal and human research [65] has shown that chronic stress alters the brain’s strategic areas, such as the hippocampus, prefrontal cortex, and amygdala. The first two areas undergo a process of neuronal loss, becoming atrophic, while amygdala increases dendritic arborization and synaptic connections, becoming hypertrophy. Hippocampal atrophy is also influenced by the blockade of neurogenesis caused by chronic stress. In fact, it is now well established that the brain produces new nerve cells in the course of learning, especially of new data. Hippocampus and prefrontal cortex atrophy, on the one hand, and amygdala hypertrophy, on the other, weaken cognitive function while increasing emotional hyperactivity. Therefore, chronic stress (from various sources, such as adverse life events, loneliness, trauma, and abuse) has been associated with brain network dysregulation and is thought to be a relevant psychiatric risk factor for depression, anxiety, post-traumatic stress disorder (PTSD), and addiction [7,66].

Growing early life research confirms that stressful experiences in the first 1000 days can result in neurobiological, cognitive, and immunological alterations that can persist in adult life, increasing the risk of psychiatric (depression, anxiety, and psychosis) and immune disorders (asthma, allergy, inflammatory diseases, and susceptibility to viral infections and oncological diseases) [67,68,69]. Some recent research has pointed out the possibility of a transgenerational transmission of imbalances in the early stages of life [70]. Epigenetic mechanisms, which we describe below, have been suggested to explain the relationship between early childhood adversity and adult health and also the transgenerational transmission of risk factors.

#### 2.2.4. The Immune System

As already mentioned above, stress is a physiological response of the body—multi-systemic and integrated—to any need of either a biological (infections or other) and mental (emotional and cognitive) nature. This is a response that, in the short term (acute stress), promotes dynamic phenomena of organism adaptation to the most varied environmental conditions, but that, if it occurs too frequently and/or for long periods (chronic stress), can have long-lasting dysfunctional effects.

Several meta-analysis studies have shown that acute stress increases IL-1β, IL-6, TNF-α, and IL-10, while it does not increase CRP, IL-4, and IL-5 [71,72], and has an activating effect on immune cells, both for natural and adaptive immunity, even in those that respond well to a viral infection, i.e., natural killers and cytotoxic T cells (Th1). In contrast, chronic stress has immunity imbalance effects, with a suppression of anti-viral circuit and an increase in natural immunity-dependent inflammatory markers.

Animal and human studies have shown that chronically stressed individuals, such as caregivers, display increased blood CRP levels and higher NF-kB activity in circulating monocytes [73]. Seminal studies by Irwin and Cole [74,75] established that life’s adversities and chronic psychosocial distress are typically associated with a hyperactivation of several proinflammatory transcription factors (i.e., NF-kB/Rel and GATA-family), an impairment of GR expression (thereby altering stress response and impairing the regulation of inflammation), and a decreased expression of anti-viral and antibody-related genes. This immune-dysregulated pattern is called “conserved transcriptional response to adversity” because it tends to stabilize across time and has been found in a wide series of life adversities, including low socioeconomic status, social isolation, and breast cancer recurrence. In contrast, prosocial engagement and non-stressing caregiving reduce inflammatory gene expression and increase anti-viral response [76,77].

The relationships between emotional distress and inflammation were also highlighted in the course of the COVID-19 pandemic [78]. Alterations in the mental status (i.e., anxiety and depression) present in COVID-19 inpatients were related to higher levels of inflammatory cytokines [79] and, in particular, showed a clear immune dysregulation recognized from the neutrophil/lymphocyte ratio, which was higher, while IL-10, major anti-inflammatory cytokine, and total lymphocyte counts were lower, compared with not having psychological symptoms [80].

Further evidence comes from research on psychiatric patients. COVID-19 hospitalized psychiatric patients versus patients with no mental disorders showed a death rate of 8.5% (vs. 4.7%) and a hospitalization rate of 27.4% (vs. 18.6%), thus showing an increased risk of infection and mortality in people with mental disorders. Depression and schizophrenia were the mental conditions at major risk [81].

## 3. Mental States and Molecular Biology

### 3.1. Epigenetics as a Main Pathway

Brain–immune cross-talk, as described above, is deeply influenced by mental states and psychosocial factors. Today, we are able to complete the psychosomatic medicine’s research program, which was initiated in the late 1930s by Franz Alexander [82] and developed in the 1970s by George Engel [83]. We can now document the molecular basis of psyche–brain–body relations and highlight the mechanisms that correlate stress, emotions, and mental and social status with the cellular machinery. We examine some examples herein, but first, we need to briefly overview the theoretical and experimental biological innovations to explain how the psychic world may become molecular biology with the principles of epigenetics.

Biological sciences are the engine of a revolution of historical importance. In place of the reductionist and determinist paradigm, a new paradigm has emerged that sees the genome no longer as the headquarters in giving in absolute autonomy instructions to the body, but as an adaptive device that responds to environmental needs by regulating gene expression. Research in the field of epigenetics vastly increased at the turn of the century, but it is an old line of research, which is a contemporary alternative to the research that dominated biology over the whole second half of the twentieth century. Epigenetics has been promoted since the early 1940s by research and publications of the British biologist, Conrad Hal Waddington, a contemporary to Francis Crick and Jacques Monod, but is divergent on the concept of the genome’s role. According to Monod, the DNA “is the fundamental invariant that gives instructions”, and “it is impossible to conceive any mechanism able to transmit any instruction to the DNA” [84], an impossibility that Crick called “the centrale dogma of molecular biology” [85]. In contrast, Waddington, studying how the genotype produces the phenotype in the context of development, concludes: “The parent couple gives to the offspring a set of potentialities, not a set of ready-made characteristics” [86]. In recent decades, the definition of epigenetics has been specified several times: “the study of molecules and mechanisms that can perpetuate alternative gene activity states in the context of the same DNA sequence” [87] or “the mechanisms enabling one genome to be programmed in many ways, resulting in diverse stable profiles of gene expression in different cells and organs in the body” [88]. However, the concept of cellular genome adaptative changes taking place in response to environmental stimulation is conserved as an epigenetical adaptive that can be either physiological or pathological.

A peculiar character of the epigenetics markers, including DNA methylation, histone modifications, and non-coding RNAs, unlike genetic mutations, can be reversible and inheritable. The reversal of such changes can be attempted using various strategies: behavioral, i.e., nutrition [89]; psychological, i.e., psychotherapy and body–mind therapy [90,91]; and pharmaceutical, i.e., “epidrugs” for cancer [92].

Inheritance can be mitotic and meiotic [93]. The former enables the stability of tissue renovation, but it also enables the maintenance and possible transmission of the functional (or dysfunctional) structure of a cell and thus its epigenetic marker (epigenome). The latter, meiotic inheritance, refers instead to the possibility of epigenetic markers being passed on to offspring. It can be intergenerational or transgenerational, that is, from parents to children or across generations [94].

### 3.2. Early Life Adversities Molecular Markers

Starting from 1976, the first results were published of a study regarding the children of the “Hunger Winter” in Holland during the Second World War, i.e., on young people born from pregnant mothers between November 1944 and April 1945, when the German occupation of the Netherlands, including Amsterdam, had reduced food supply to the population to 400–800 calories per day. The offspring of these women who had suffered hunger, especially in the first three-month period of their pregnancy, were born with a lower-than-normal birth weight. Thirty-five years later, the researchers were able to record, in this group of children born in hunger conditions, now adults, an increase in the incidence of various psychiatric disturbances, including mood issues (anxiety and depression), anti-social personality disorders, and schizophrenia; an accelerated decline in cognitive functions at the age of 56–59 years; as well as an increase in the typical disturbances linked to low birth-weight, such as diabetes, obesity, and cardiovascular problems [95,96]. What could be a possible explanation for this?

In a project carried out by a group of epidemiologists at Leiden University Medical Center, the Netherlands, in 2008, it was demonstrated for the first time that the children born in hunger conditions, 60 years later, presented an alteration of methylation of the gene controlling the synthesis of IGF-2 [97], i.e., the insulin-like factor of type 2 which regulates the growth of the fetus and which, if it is hypoactive, determines a low weight at birth. Several years after, genome-wide changes in adult DNA methylation were found in the subjects prenatally exposed to the Dutch famine [98].

The above-mentioned research provides evidence that, during the first stages of life, the environmental conditions can cause epigenetic changes that persist for the remainder of the individual’s life.

In 2004, a McGill University research group published a work which described a major change, since for the first time it was demonstrated, using epigenetics, that a behavior leaves its enduring sign on cerebral biology [99]. Young rats raised by “negligent mothers” (i.e., lacking in the common licking and grooming care towards their young), with respect to others raised by accurately “caring” mothers, presented a hyper-methylation at the level of the cytosine and the histones of the receptor gene promoter for the glucocorticoids (GR) of the hippocampus. The animals raised by negligent mothers, during the course of their development, presented an alteration of the stress response and, most importantly, the females of the animals presented the same epigenome as the mother and, therefore, reproduced the same uncaring attitude towards their own offspring. A central infusion of a histone acetylase inhibitor removed the differences in the histone acetylation, in DNA methylation, in the expression of the receptor for glucocorticoids (GR), and in the response to the HPA axis to stress. Lastly, the fact that it is maternal behavior that induces the epigenetic marking and not a genetic predisposition is demonstrated by the fact that when offspring born to caring mothers were placed in the cages with uncaring mothers, the offspring hypothalamus showed methylation of the gene for GR, and these animals accordingly behaved in the same way as the young born to negligent mothers. Thus, this research shows that maternal behavior changes the offspring gene status, and it is reversible.

Studies on humans in recent years are confirming what has been documented in animals. A meta-analysis found a significant correlation between psychosocial maternal stress and offspring methylation at a specific CpG site located in the exon 1F of the human glucocorticoid receptor gene NR3C1 [100]. Exon 1F is equivalent to 17 hyper-methylated rats that receive poor maternal care.

Stress during pregnancy is another notable line of evidence on the epigenetic modulation of fetus development. It is associated with an inflammatory internal environmental that epigenetically marks the neuroendocrine stress axis and some key molecules of the fetus [101].

Maternal adversities, such as stress life events, low social status, anxiety, depression and malnutrition, correlate with alterations in the DNA methylation in offspring genes, including NR3C1, BDNF, SLC6A4, OXTR, and 11 β-HSD-2 [102,103].

11-beta-hydroxysteroid dehydrogenase (11 β-HSD-2) converts maternal cortisol to less active cortisone, and its epigenetic alteration exposes the fetal brain to an excess of cortisol with possible extensive long-term destructive effects. Brain-derived neurotrophic factor (BDNF), serotonin transporter (SLC6A4), and oxytocin receptor alteration (OXTR) affect major brain systems with a possible expression of related mental disorders.

Pregnant women experienced high levels of stress during the COVID-19 pandemic. Anxiety and depression were the most frequent disorders. According to research based on a large sample, the increase in depressive symptoms was 33% and that of anxiety symptoms was 47% compared to the pre-pandemic period. The researchers found significant relationships between prenatal maternal distress and infant amygdala–prefrontal micro-structural and functional connectivity alteration. Thus, distress in pregnancy is related to brain changes in 3-month-old infants [104]. According to a meta-analysis, distress among pregnant women during natural disasters (ice storms and cyclones) is related to 10-year-old children with worse cognitive, motor, socio-emotional, and behavioral outcomes [105].

The immune system is also dysregulated. Some research on pregnant women during the Quebec ice storm in 1988 has found a relationship between the maternal stress level, measured soon after disaster, and the total count of CD4+ lymphocyte reduction, and the increase in TNF-α, IL-1β, IL-6, IL-4, and IL-13 levels in 13-year-old children [5]. The inflammatory cytokine enhancement and Th2 shift (through increases in IL-4 and IL-13) explain, via epigenetic signature, the increase in asthma incidence in this child population [106]. According to pediatric research, there is mounting evidence that prenatal stress and mental disorders alter immune epigenetic profiles and subsequent function in exposed offspring [107].

### 3.3. Loneliness

Being isolated, with few social ties, or even feeling alone, despite living in a suitable family and social context, is probably the most painful and even most dangerous psychic condition for human health. People who feel alone are in a permanent state of alertness, are afraid of others, are afraid of judgment from others, are afraid of being rejected, feel guilty, or have no prospects. Recently, major studies on the effects of isolation on the human immune system have reviewed these elements [108]. Isolation and social exclusion, in older men, in 40-year-old males and females, and in children, are associated with: (1) a typical psychological profile, characterized by anxiety, fear of receiving negative evaluations from others, and extreme sensitivity to rejection; (2) a strong increase (doubling) in the levels of inflammatory markers (C-reactive protein (CRP) and interleukins); and (3) and a remarkable reactivity of the immune system to both social and natural stressors (e.g., seeing a snake attacking). Among the two types of stressors, social and natural, the first is a much more powerful stress activator than the second. However, living in a condition of social isolation exposes a person to increased inflammatory reactivity to natural stressors, such as pathogenic micro-organisms.

The immune systems of people who live and feel alone are epigenetically modified in a pro-inflammatory sense. Seminal works of Steve Cole showed upon the chronic activation of the stress system, e.g., when living in a condition of loneliness, there is an induction of a “conserved transcriptional response to adversity” (CTRA) in peripheral immune cells, dendritic cells, and monocytes in particular. This is characterized by the increased expression of proinflammatory genes (i.e., IL-1β, IL-6, and TNF-α) and the decreased expression of anti-viral- and antibody-related genes (i.e., IFNs) [76]. Immune dysregulation is particularly dangerous in a viral pandemic, such as COVID-19 [109]. An experimental study on adult macaques placed in solitary confinement for two weeks, which mimicked human lockdown, showed, already within the first 48 h, a marked reduction in all circulating immune cell populations, and the down-regulation of type I interferon (IFN) anti-viral gene expression [78].

In this regard, it should be noted that the anti-viral circuit also carries out immunosurveillance against tumors. Studies on breast and ovarian tumors from socially isolated women have documented a systematic upregulation of pro-metastatic genes that drive both epithelial–mesenchymal transition (EMT) and inflammation, which are ineffective against tumors, macrophage polarization (M2), and increases in lymphatic vessels in the tumor and the microenvironment [110,111].

Lastly, loneliness has notable effects on the brain and metabolic systems. Hippocampal dentate gyrus and plasmatic BDNF showed a significant reduction in voluntary isolation during a long Antarctic expedition [112]. A systematic review of 41 loneliness studies (over 16,000 participants) that utilized various brain imaging technologies (CT, MRI, fMRI, DTI, etc.) showed the alteration of the structure and/or function in the medial and dorsolateral prefrontal cortex, anterior insula, amygdala, hippocampus, and posterior superior temporal cortex [113]. The same systematic review documented a relationship between loneliness and increased risk for the onset of dementia, as well as an increase in biological markers (amyloid and tau burden) associated with Alzheimer’s disease. Furthermore, loneliness is related to various metabolic alterations, such as type 2 diabetes mellitus, dyslipidemia, metabolic syndrome, hypertension, and other cardiovascular risk markers [114].

### 3.4. Social Adversity and Social Inequality

Respectability is very important to us. It is a typical human socially constructed feeling. It also takes shape early in our psyche and can even be traced back to a child of 5 years old [115,116]. Feeling inadequate and ashamed are feelings common to most human beings. Usually, they are transient phenomena related to phases of life (i.e., infancy and adolescence) or conditions (i.e., college admission and job loss) or status (i.e., gender, sexual orientation and race), which are reduced by gratification and social support. They can, however, be a personality trait or the sign of a traumatic and socially disadvantaged condition. A long series of studies in the 1990s on homosexuals with HIV showed their shame, which led to them concealing their sexual identity, and also caused self-depreciation, increased inflammation, and predicted subsequent viremia [117]. A study on homosexual men in Los Angeles has examined the relation between homophobic victimization experience and conserved transcriptional response to adversity (CTRA, see above) and showed that CTRA gene expression was increased by 3.1-fold in homosexual men who experienced homophobic victimization [118]. Research has, more recently, been extended to so-called sexual minorities (homosexual, transgender, non-binary, etc.). A systematic review has shown that acute exposure to a minority stressor revealed immediate changes in blood cell counts, and the condition of minority stress was related to the development of subsequent respiratory infections related to immune gene expression [119].

Even in healthy subjects, evoking feelings of shame, and also of body shame, causes an increase in the TNF-α [120] and cortisol [121]. Therefore, a troubled couple relationship, which is highly conflictual even if partners are young and healthy people, alters the stress and immune systems, according to Janice Kiecolt-Glaser’s 30-year study. A longitudinal study on 90 newlywed couples, followed for 10 years, showed that the epinephrine and norepinephrine levels of the couples that were more conflictual in the first year of marriage were elevated compared to couples with marriage satisfaction, and elevations of catecholamines were stable and were not a trivial transient response to conflict [122]. Norepinephrine has potent effects on the immune system, and inflammatory and anti-inflammatory receptors (α- or β-adrenergic receptors, respectively), but the chronic activation of the sympathetic system, lowering the anti-inflammatory action of the vagus nerve, has the net result of an inflammation increase. In fact, the rise in the norepinephrine levels of newlyweds correlates with studies linking divorce to increased inflammation [123] and shorter telomeres [124]. According to Kiecolt-Glaser, depression due to conflictual marriage provides a major pathway to immune dysregulation and poor health, including a higher incidence of obesity and sleep disorders. Kiecolt-Glaser also argues that women are more negatively affected compared to men, which is also due to having more disadvantaged social relations than men.

Gender, race, sexual orientation, and socioeconomic position are embedded in human biology. Recently, extensive research has focused on the relationship between socio economic position (SEP) and inflammation. The study, which was conducted over 55 years (1958–2013) involving 23,000 people from three European countries, found an inverse relation between SEP and CRP (as inflammation marker), and that participants with a lower SEP have higher levels of inflammation (CRP) [125].

Such findings were confirmed and enhanced in a larger study funded by the European Commission Horizon 2020 program, i.e., Lifepath research pooled data on up to 1.7 million participants of longitudinal cohort studies from Europe, USA, and Australia [126]. According to the Lifepath study, low SEP was associated with 2.1 years of life lost (YLL) between the ages of 40 and 85 years. More importantly, SEP is a primary risk factor among the traditional and confirmed major risk factors, i.e., smoking, diabetes, and physical inactivity. The years of life lost due to lower SEPs are greater than those lost due to hypertension, obesity, and alcohol abuse. Psychosocial stress associated with low SEP involves inflammatory responses, impaired immune function, and the epigenetic acceleration of aging, according to the authors. Lifepath research was able to confirm the relationship between psychosocial stress and damage to health in adults, using different methods, including the assessment of the allostatic load, inflammation, and biological aging through epigenetic parameters (DNA methylation). Standard allostatic load was extended to 16 blood-derived biomarkers signaling the activity of 6 physiological systems (including cardiovascular, inflammation, metabolic, endocrine systems, and the functions of both the liver and kidney), as defined by the biological health score (BHS). High BHS correlates linearly with more disadvantaged social groups. Therefore, CRP and 28 other inflammatory proteins, investigated with various methods and through gene expression, correlate with low SEP. Lastly, the Lifepath study used established epigenetic clocks as markers or predictors of accelerated aging. The epigenetic clock measured the difference between biological and chronological ages through full DNA methylation. The findings showed an age acceleration in disadvantaged social groups.

### 3.5. Depression, and Other Psychiatric Diseases

The vicious circle between stress, inflammation, and depression, in the last 25 years, has been widely analyzed even in molecular detail [127,128]. A significant proportion of people with depression have clear signs of inflammation in their blood, with an increase in C-reactive protein and pro-inflammatory cytokines [129]. A meta-analysis of the mean differences and variability in 5166 patients and 5083 controls showed that the levels of CRP, IL-3, IL-6, IL-12, IL-18, sIL-2R, and TNF-α were significantly higher in patients with depression [130]. In peripheral blood mononuclear cells, pro-inflammatory cytokine values between people with depression and controls were investigated by another study, with striking results. For example, the difference in the amount of IL-6 in the group with depression was ninety-fold greater than in the controls (978.1 pg/mL versus 11.1 pg/mL) [131]. Using CRP > 3 mg/L as a cut-off value, approximately 40% of cases with depression had increased immune cell counts, increased inflammatory proteins and increased symptom severity scores, compared to the remaining 60% of cases without inflammation. However, the multivariate analysis of patients with depression could document that the proportion of cases with depression associated with inflammation was higher and underestimated by the CRP cut-off. This research highlights the need for a more in-depth and refined study on the different forms of inflammatory depression, which is a frequent phenomenon in the course of depressive disorder. However, inflammation occurs not only as a result of stress, but also from individual (obesity, inflammatory diseases (such as cardiovascular), and autoimmune diseases) and collective (pollution) conditions and behavior (inflammatory diet, sedentary lifestyle, and the use of medicines and drugs). There is a bidirectional relationship between cardiovascular disease and depression, sharing immune dysregulation and inflammatory mediators. The current view of coronary heart disease has deeply changed [132,133], as atherosclerosis is no longer considered a simple lipid storage disorder but a systemic inflammatory disease. Chronic depression has been identified as an important risk factor in people who have already had a heart attack. It is possible to explain the link between a depressed mental state and reinfarction by keeping in mind that cardiac activity is regulated by the brain via the autonomic nervous system (neurocardiac axis).

Moreover, several psychiatric diseases are connected with inflammation. In people with obsessive compulsive disorder (OCD), high levels of the main inflammatory cytokines (IL-2, IL-4, IL-6 and TNF-a) were found in the circulating blood, as well as a hyperactivation of the stress system with increased ACTH and cortisol [134]. Recently, research documented epigenetic hypomethylation in all brain areas, but the nucleus accumbens presented a predominant hypomethylation pattern in the post-mortem of patients with OCD compared to the controls [135]. Moreover, the relationship between obsessive compulsive disorder and inflammation is effectively evidenced by new syndromes identified by pediatric neuropsychiatry, such as pediatric autoimmune neuropsychiatric disorders associated with streptococcal infection (PANDAS), a neuropsychiatric syndrome in children that was initially thought to be necessarily associated with a streptococcal infection, whereas it was subsequently found that the infection may also not be present, even if there are signs of inflammatory alterations. Hence, the new name pediatric acute-onset neuropsychiatric syndrome (PANS) comprises several syndromes, which are mostly characterized by the typical symptomatology of obsessive compulsive disorder and closely related diseases, such as Tourette’s disorder and other tic disorders [136].

The relationships between social stress and schizophrenia, in recent years, have been well documented. Being an immigrant involves a relative three-fold increase in the risk of developing schizophrenia, compared with the average; such risk becomes four-fold if the immigrant is identified as part of a minority group. Similarly, it has long been known that the risk of schizophrenia is almost doubled for those born in cities, and it is also known that city life is much more stressful than that in small towns [137]. Experimental research on patients with schizophrenia has documented that these subjects are much more sensitive than controls to normal stressful life events and that under stressful stimuli, even of medium severity, and has shown an increase in the symptoms of the disease [138]. Recently, a very similar stress response dysregulation in people at clinical high risk for psychosis was documented [139]. Stress has effects on the dopaminergic system and on the other related systems (glutamate in particular). A systematic review [140] and a recent study documented that high levels of PCR and pro-inflammatory cytokines and chemokines (MCP-1) [141] in patients’ blood correlate with a cognitive deficit, which was measured with a specific scale. Patients who, in addition to having elevated inflammatory indices, have antibodies against the herpes simplex virus (HSV-1), showed deeper negative cognitive effects. Finally, patients experiencing their first episode of psychosis showed a strong increase in MCP-1 chemokine and a significant parallel decrease in working memory and executive functions.

### 3.6. Stress, Mental Condition and Vaccine Effectiveness

For three decades, Ronald Glaser and Janice K. Kiecolt-Glaser’s lab at Ohio State University College of Medicine has been studying the effects of stress on vaccine efficacy (for a review, see [142]). Studies starting in 1992 on college students vaccinated for hepatitis B showed that academic session stress negatively impacted the antibody response to the vaccine.

In subsequent years, Alzheimer’s disease caregivers vaccinated against flu were tested. Interestingly, only 38% of them responded to the vaccine; in fact, they had high levels of cortisol and a deficit of the immune adaptive response. The deficit persisted even after the death of the spouse who required care.

Studies on the spouses and children of people with Alzheimer’s disease, i.e., subjects directly involved in caregiving, have confirmed the weak immune response to the vaccine. Results were also replicated by research on parents of children with neurodevelopmental disorders. Furthermore, double-blind studies compared with a placebo have shown that a stressful condition at the time of vaccination increases the inflammatory response, which can contribute to post-vaccination adverse effects and the weakening of the immune memory.

Last, but not least, inflammation accompanying depression and other mental disorders worsens vaccine response, as documented by various studies, and summarized and analyzed by the excellent review by J.K. Kiecolt-Glaser’s group, above citated.

Therefore, it can be assumed that psychological interventions in the COVID-19 pandemic not only relieve psychic suffering, but can significantly contribute to immunological resistance against SARS-CoV-2 infection diffusion and to the population’s resilience possibilities [143].

### 3.7. Epigenetic Signature Reversion and Inflammatory Mediator Regulation by Psychological and Body–Mind Interventions

When psychotherapy works, it also improves the inflammatory state.

Prolonged exposure therapy (PET) is the standard treatment of post-traumatic stress disorder (PTSD). In responders to psychotherapy, a significant reduction in the methylation of the GR gene (NR3C1) was identified. This gene methylation reduction regulates cortisol, improving production both at baseline and under stress tasks [144,145].

A systematic review [146] investigated the role of psychotherapy, in particular, cognitive behavioral therapy, in chronic inflammation reduction in patients with depression. Although the studies were somewhat heterogeneous, most research has shown a clinically significant decrease in at least one inflammatory biomarker within a wide range of markers examined, such as the serum concentration of cytokines (TNF-α and IL-6), the expression of nuclear transcription factors (NF-kB), and the immune cell count and activity of innate and acquired immunity (natural killer cells and T lymphocytes).

A recent study, conducted on a group of patients with moderate depression, documented a significantly higher level of chemokines, compared with controls without depression. Chemokine levels after the online psychotherapy intervention had significantly dropped [147].

Psychosocial interventions, including cognitive behavioral therapy and other forms of psychotherapy, are associated with a reduction in inflammatory markers and an increase in anti-viral immunity, according to a recently published systematic review and meta-analysis of 56 RCT with 4060 participants [148].

Studies conducted over the past two decades have shown that mind–body techniques, including meditation, yoga, tai chi, and qi gong, based on ancient traditions, are effective practices still used today to moderate the effects of stress on the immune system. Mind–body techniques, including HRV biofeedback [149] and neurofeedback [150], regulate the neuroimmune system, thanks to the modulation of the brain areas involved in the stress response control (prefrontal, cingulate cortex, amygdala, and hypothalamus), increasing parasympathetic activity and reducing sympathetic discharge. These effects can directly influence the gene expression of immune cells suppressing the signaling of NF-kB, and consequently reduce the inflammatory state.

Two controlled psychoneuroendocrinoimmunology meditation (PNEIMED)-based studies, in healthy middle-aged and young volunteers, showed a reduction in salivary cortisol under basal and stressful conditions [151,152].

A review by Bower and Irwin, which touched on 26 different studies [153], analyzed the effects of mind–body techniques on some inflammatory markers, such as PCR, noting that tai chi, qi gong, and yoga are more likely to reduce their levels; it is important to underline that at least one half of the results are derived from studies conducted on groups of people with pathologies. Research conducted on breast cancer survivors after a three-month follow-up showed that intensively practicing yoga can reduce the production of TNF-α, IL-1, and IL-6 by monocytes. Additionally, the practice of tai chi reduces the expression of TNF-α and IL-6 in monocytes of people affected by insomnia.

It is worth noting that meditation and psychological interventions together (COBMINDEX) showed efficacy in patients with Crohn’s disease, both increasing wellbeing and decreasing inflammatory markers connected with the disease [154].

Moreover, a meta-analysis [91] highlighted that the practice of meditation is associated with a general profile of expression genes characterized by a significant under-regulation of genes and pro-inflammatory signaling pathways, with NF-kB as the key factor.

Additionally of interest is the study of the immunoregulatory effects of qi gong, which not only lowers the inflammatory component of innate immunity, but also enhances B- and T-cell activity [155].

Clinically, high-quality studies are growing, documenting high and moderate efficacy for some mind–body integrative interventions, such as mindfulness for schizophrenia, attention deficit hyperactivity disorder (ADHD) and PTSD [156], and biofeedback and neurofeedback for depression [157] and ADHD [158], although more RCTs are needed [159].

Collectively, the results of such research show the potential mechanistic pathways mediating the transduction of psychological interventions (psychotherapy, meditation, and body–mind techniques) into patterns of gene expression and the regulation of the inflammatory processes.

## 4. Conclusions and Perspectives

Here, we reviewed the major evidence on the mutual communications between the psyche and biological systems describing the pathways linking the psyche–brain–immune system and the molecular mechanisms by which stress and mental conditions modulate genomic expression.

However, there is still a long way to go. We point out only two fields of research that can greatly enrich our knowledge of the relationship between the psyche and biological systems: first, the knowledge of the vast world of microbiota and, therefore, of its influences on psychic life and mental disorders; and second, epigenetic research, which is still in its infancy, although the epochal significance of the epigenetic revolution is indisputable, and has not yet developed its full considerable potential for knowledge on the molecular translation of mental states in the brain. Future scientific acquisitions will likely clarify the complex interplays between environmental, mental, and biological dimensions in more detail which we have mentioned in this review.

Our review has a fundamental limitation: we have not carried out a systematic review. Therefore, some topics have not been considered, including the role of sleep and dream activity in the construction of memory and mental states and their involvement in the regulation of the immune response. In order to grasp a deeper understanding on these issues, we point out some excellent reviews [160,161].

The current evidence, however, is sufficient to shape a new scientific paradigm: psychoneuroendocrinoimmunology (PNEI), which studies the human being as a whole, integrating biology and psychology.

It is not a reductionist materialist model, since the psyche is not an epiphenomenon without a history; a language; its own modalities of cultural transmission; its relative autonomy; and, above all, the ability to act, unconsciously and consciously, on other systems. It is not a spiritualist model either, because it does not presuppose a mysterious or divine origin of the psyche, nor its alienation from the other systems of the human network.

It is indeed a scientific model that is, therefore, capable of producing knowledge in the biological, medical, and psychological fields, and in philosophical and anthropological endeavors [162].

This scientific paradigm can deeply change medical and psychological sciences and clinical practice, integrating psychology and biomedicine, because the disciplines are artificially divided, which is not the reality [163].

Therefore, every patient, when seeing a doctor, a surgeon, a psychologist, or another therapist, should receive an integrated diagnosis, i.e., an analysis considering and combining examinations and biological and psychological evaluations, in the context of the reconstruction of the biography of the subject, that covers the main events of life and not simply those concerning “sanitary health”, as is practiced in traditional medicine.

At the same time, the therapist should know the effectiveness, limits, and risks of each of the possible therapeutic proposals and, therefore, know that not only drugs; however, social support, psychotherapy, meditation, physical exercise, and body manipulations can also affect care. Indeed, their combination, adapted to the individual patient, may have surprising synergistic effects.

This entails a fundamental change in the organization of knowledge and its transmission. In our opinion, it is necessary to highlight the common foundation of the so-called life sciences and human sciences, since they deal with the two levels of organization of the human condition, the biological one and the historical–social one [164]—levels that are closely intertwined, not separable, and therefore, cannot be studied in isolation from each other.

This unity of knowledge, which was powerful in the ancient world, and renewed by the Renaissance, remaining in Europe until the mid-nineteenth century, needs to be rebuilt with new generations of “bilingual scientists and philosophers” (in a broad sense), to resume an effective expression of Thomas Kuhn [165] that can break down linguistic, doctrinal, specialist, and caste barriers.

## Data Availability

Not applicable.
